# Troponin I levels in permanent atrial fibrillation—impact of rate control and exercise testing

**DOI:** 10.1186/s12872-016-0255-x

**Published:** 2016-05-04

**Authors:** Anja Wiedswang Horjen, Sara Reinvik Ulimoen, Steve Enger, Jon Norseth, Ingebjørg Seljeflot, Harald Arnesen, Arnljot Tveit

**Affiliations:** Department of Medical Research, Baerum Hospital, Vestre Viken Hospital Trust, N-3004 Drammen, Norway; Faculty of Medicine, University of Oslo, Oslo, Norway; Clinic for Medical Diagnostics, Vestre Viken Hospital Trust, Drammen, Norway; Center for Clinical Heart Research, Department of Cardiology, Oslo University Hospital Ullevål, Oslo, Norway

**Keywords:** Atrial fibrillation, Biomarkers, Exercise testing, High-sensitivity cardiac troponin I, High-sensitivity cardiac troponin T, Rate control

## Abstract

**Background:**

High-sensitivity troponin I (hs-TnI) and troponin T (hs-TnT) are moderately correlated and independently related to outcome in atrial fibrillation (AF). Rate controlling therapy has been shown to reduce hs-TnT, however the potential impact on hs-TnI levels, and whether this differs from the effects on hs-TnT, has not been investigated previously.

**Methods:**

Sixty patients with stable, permanent AF without heart failure or known ischemic heart disease were included in a randomised crossover study (mean age 71 ± 9 years, 18 women). Diltiazem 360 mg, verapamil 240 mg, metoprolol 100 mg, and carvedilol 25 mg were administered once daily for three weeks, in a randomised sequence. At baseline and on the last day of each treatment period, hs-TnI was measured at rest and after a maximal exercise test and compared to hs-TnT.

**Results:**

Hs-TnI and hs-TnT correlated moderately at baseline (r_s_ = 0.582, *p <* 0.001). All drugs reduced both the resting and the peak exercise levels of hs-TnI compared with baseline (*p <* 0.001 for all). The decline in resting hs-TnI and hs-TnT values relative to baseline levels was similar for all drugs except for verapamil, which reduced hs-TnI more than hs-TnT (*p =* 0.017). Levels of hs-TnI increased significantly in response to exercise testing at baseline and at all treatment regimens (*p <* 0.001 for all). The relative exercise-induced increase in hs-TnI was significantly larger compared to hs-TnT at baseline (*p <* 0.001), on diltiazem (*p <* 0.001) and on verapamil (*p =* 0.001).

**Conclusions:**

In our population of stable, permanent AF patients, all four rate control drug regimens reduced hs-TnI significantly, both at rest and during exercise. The decline in hs-TnI and hs-TnT levels associated with beta-blocker and calcium channel blocker treatment was similar, except for a larger relative decrease in hs-TnI levels following verapamil treatment.

**Trial registration:**

www.clinicaltrials.gov (NCT00313157).

## Background

Atrial fibrillation (AF) confers an independent risk for stroke and death [1, 2]. High-sensitivity cardiac troponin assays permit measurements of very low levels of circulating troponins, and have revealed a low-level, chronic troponin release in AF populations [3–5]. Minor elevations in cardiac troponins below the 99^th^ percentile upper reference limit are associated with cardiovascular morbidity and mortality in AF [6–9], and persistent elevations indicate worse prognosis than transient elevations [10]. As cardiac troponins emerge as a prognostic biomarker in AF, it is of paramount importance to recognise the factors influencing on troponin levels. An imminent and unsolved issue is to what extent the different treatment modalities in AF are capable of modulating cardiac troponin levels.

In the Rate Control in Atrial Fibrillation (RATAF) study, beta-blockers and calcium channel blockers reduced high-sensitivity troponin T (hs-TnT) levels significantly [11], supporting evidence of an association between heart rate and troponin levels in AF [12]. The RATAF study also revealed an exercise-induced increase in hs-TnT levels [11]. A rise in cardiac troponins in response to exercise testing has been demonstrated in patients without evidence of myocardial ischemia [13, 14], suggesting alternative mechanisms for troponin release other than cardiac cell death.

High-sensitivity troponin assays have revealed differences in sensitivity and specificity between high-sensitivity troponin I (hs-TnI) and hs-TnT with potential clinical implications [15, 16]. The two molecules appear to be only moderately correlated in AF, with hs-TnI being more associated with cardiovascular diseases and AF burden, and hs-TnT with age, male sex and diabetes [17]. These observations indicate that factors influencing low-level, chronic troponin elevation may differ between hs-TnI and hs-TnT. At present, it is unknown whether rate control therapy and exercise testing in an AF population affect the two troponin subtypes differently.

Accordingly, the objectives of the present study of patients with permanent AF were first to assess the impact of four rate-reducing drugs on hs-TnI levels at rest and after a maximal exercise test using a high-sensitivity assay and second to compare the results with those obtained using a high-sensitivity assay for troponin T.

## Methods

### Study design

**Fig. 1:**
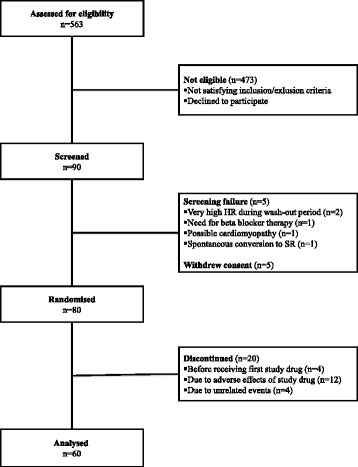
Flow chart of the study. HR, heart rate; n, number of patients; SR, sinus rhythm

The present study was a substudy of the RATAF study, in which four different once-daily drug regimens for rate control in permanent AF were compared in a prospective, randomised, investigator-blind, crossover study [18]. A flow-chart of the study is presented in Fig. 1. Briefly, patients age >18 years with stable, permanent AF without heart failure (clinical or radiological signs of congestive heart failure and/or reduced ejection fraction) or known ischemic heart disease were included. Patients who were treated with rate-reducing drugs at the time of inclusion had a wash-out period of two weeks before baseline evaluation was performed. The patients on digitalis were instructed to discontinue this drug, and did not start the wash-out period until digitalis was undetectable in serum. After baseline evaluation, the participants were randomised through a computer-generated block randomisation list to receive all of the following drug regimens for at least three weeks in a randomised cross-over design: (I) metoprolol slow-release tablets 100 mg o.d. (AstraZeneca), (II) diltiazem sustained release capsules 360 mg o.d. (Pfizer), (III) verapamil modified release tablets 240 mg o.d. (Abbott), and (IV) carvedilol immediate release tablets 25 mg o.d. (Roche/HEXAL). The patients were randomised from May 2006 to June 2010. The investigator was blinded with regard to study drug sequence, whereas for practical reasons the participants were aware of the drug assigned. Compliance of the drug regimen was assessed by pill count after each drug period. Before starting the first treatment, and on the last day of each treatment period, serum samples were collected at rest, at peak exercise and 15 min after exercise termination. The RATAF study was approved by the Regional Ethics Committee and the Norwegian Medicines Agency, and all patients signed informed consent in accordance with the Helsinki

Declaration. The RATAF study was registered at www.clinicaltrials.gov (NCT00313157) at 10th of April 2006.

### Baseline evaluations

All patients underwent clinical examination before entering the study. Left ventricular systolic function was assessed with echocardiography. Echocardiographic measurements were averaged over five cardiac cycles, if possible in a phase with close to normal heart rate and relative regular R-R intervals. Lung function was assessed by spirometry and diffusing capacity.

### Exercise test

The cardiopulmonary exercise tests were performed on a bicycle ergometer (Ergoline 800, Bitz, Germany) in accordance with American College of Cardiology/American Heart Association guidelines [19]. Details have been published previously [20]. The expected peak oxygen uptake was calculated for each patient. Individual protocols were chosen based on age, gender and weight, with the aim of reaching each patient’s estimated maximal performance in 8 to 12 min. The patients were tested with their individual protocol every time, preferably on the same time of the day. Oxygen consumption, carbon dioxide production, and respiratory exchange ratios were measured continuously during exercise by an automated gas exchange system (Vmax Spectra, SensorMedics, CA, USA). Patients were encouraged to maintain a pedaling rate of at least 60 per minute and continue until exhaustion. A physician and a technician blinded to the patients’ treatment were present during all tests.

### Arrhythmia-related symptoms

Arrhythmia-related symptoms were assessed using a self-administered questionnaire: The symptom Checklist—Freqency and Severity (SCL) in Norwegian translation [21, 22]. The SCL questionnaire rates the frequency (from 0 to 4) and severity (from 1 to 3) of 16 symptoms potentially associated with AF, with higher scores representing worse symptoms.

### Troponin levels

Blood samples for hs-TnI and hs-TnT analysis were drawn after 30 min of rest in the supine position before the exercise test, at peak exercise and 15 min after exercise termination. Serum was prepared within one hour by centrifugation at 2000 x *g* for 15 min at room temperature. Aliquots were then stored at −70 °C and later analysed in one batch. The samples had been through one freeze-thaw cycle before the analyses of hs-TnT and two freeze-thaw cycles before the analyses of hs-TnI. In patient samples, cardiac troponins are reported to be stable at −70 °C and through repeated freeze-thaw cycles [23–25].

Hs-TnI levels were determined using the ARCHITECT_STAT_ high sensitive troponin I assay (Abbott Laboratories, Abbott Park, Illinois, USA), with a limit of blank of 0.7 ng/L, a limit of detection of 1.2 ng/L and a limit of quantification of 5.0 ng/L. The coefficients of variation in our laboratory were 11.7 % for hs-TnI = 2.5 ng/L, 6.4 % for hs-TnI = 28.5 ng/L and 5.2 % for hs-TnI = 178.1 ng/L. The 99th percentile upper reference limit for healthy individuals is 23 ng/L for the entire reference population (36 ng/L in men and 15 ng/L in women) [26].

Results with regard to hs-TnT and N-terminal pro-B-type natriuretic peptide (NT-proBNP) have been published previously, and are included in this article as reference [11, 20]. Hs-TnT levels were analysed on the Cobas e411 analyser using the Roche high sensitive Troponin T assay (Roche Diagnostics, Basel, Switzerland) with a limit of blank of 3.0 ng/L, a limit of detection of 5.0 ng/L and a limit of quantification of 13.0 ng/L. The coefficients of variation in our laboratory were 5.0 % for hs-TnT = 13.1 ng/L, 5.5 % for hs-TnT = 30.4 ng/L and 1.4 % for hs-TnT = 85.2 ng/L. The 99th percentile upper reference limit for the entire reference population is 14 ng/L (15 ng/L in men and 10 ng/L in women) [27]. NT-proBNP was assessed using the Elecsys proBNP sandwich immunoassay on an Elecsys 2010 (Roche Diagnostics, Basel, Switzerland).

### Statistical analysis

Categorical variables are given as frequencies (%) and continuous variables are given as mean ± standard deviation (SD) for normally distributed variables, whereas median (25^th^ percentile,75^th^ percentile) is given for variables not normally distributed. P-values from multiple comparisons between treatments were Bonferroni adjusted. Group comparisons of continuous variables were tested by Student *t* test or the Mann–Whitney *U*-test depending on distribution. Categorical data were compared by the chi-square test or Fischer’s exact test where appropriate. The impact of continuous clinical variables on hs-TnI and hs-TnT was analysed using Spearman correlation coefficient, denoted r_s_. Variables associated with logarithmically transformed hs-TnI and hs-TnT were examined using univariate and multivariate linear regression analysis. Variables related to troponin levels with a *p*-value of <0.10 in univariate analyses were included in a multivariate regression model. Medications at randomisation were not included as they were thought only to reflect the disease that indicated their use. Wilcoxon signed-rank test was used to compare hs-TnI levels at rest and at peak exercise. The different treatment regimens (including baseline with no drug intervention) were compared using a linear mixed model for repeated measurements, with a random intercept for each patient. Possible carryover effects were assessed with an interaction term between treatment regimens and time periods. As this interaction term was not statistically significant, it was removed from the final statistical model. Spearman correlation coefficient was used to examine correlations between hs-TnI and hs-TnT, and a two way scatterplot with a fitted ordinary least-squares regression line was used. Troponin values were logarithmically transformed before entered into the mixed model and the linear regression model. A two-sided *p*-value of <0.05 was considered statistically significant. Statistical analyses were performed with IBM SPSS Statistics for Windows, version 21.0 (IBM Corp., New York, USA).

## Results

**Fig. 2:**
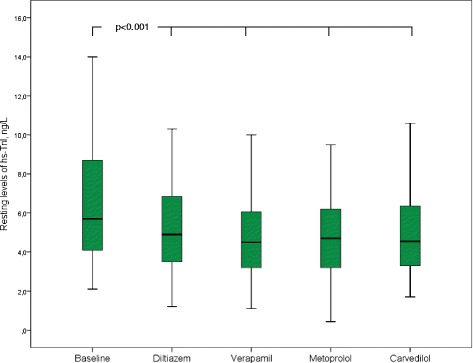
Resting hs-TnI levels at baseline and during treatments. All drug regimens reduced the resting levels of high-sensitivity troponin I compared to baseline (*p <* 0.001 for all, p-values derived from the Wilcoxon signed-rank test). Center lines show the medians; box limits indicate the 25th and 75th percentiles; whiskers extend 1.5 times the interquartile range from the 25th and 75th percentiles. Abbreviations: hs-TnI, high-sensitivity troponin I

Baseline characteristics of the 60 participants that completed the study are given in Table 1. Hs-TnI was detectable in all patients. Four of the patients (7 %) had levels above the sex-specific 99th percentile of a healthy reference population (36 ng/L in men and 15 ng/L in women). The median (25^th^ percentile, 75^th^ percentile) hs-TnI level at rest was 5.2 (3.8, 8.5) ng/L at baseline (no treatment), 4.5 (3.3, 5.9) ng/L during treatment with diltiazem, 4.1 (2.8, 5.6) ng/L on verapamil, 4.5 (3.0, 6.0) ng/L on carvedilol and 4.1 (2.7, 6.2) ng/L on metoprolol (Table 2, Fig. 2). Resting hs-TnI levels did not correlate to gender. We found no associations between baseline levels of hs-TnI and the presence of comorbidities like hypertension, diabetes mellitus, renal impairment, stroke or chronic obstructive pulmonary disease. Hs-TnI did neither correlate to NT-proBNP levels nor CHA_2_DS_2_-VASc score, which is a measure of stroke risk in patients with atrial fibrillation, with scores ranging from 0 to 9 and higher scores indicating greater risk. NT-proBNP correlated to baseline hs-TnT (r_s_ = 0.331, *p <* 0.001). In multivariate analysis, older age was associated with higher hs-TnT levels (*p =* 0.006) [11], whereas no association was found between age and hs-TnI.

**Table 1:** Baseline characteristics

Variable	*N =* 60
Age, years	71 ± 9
Gender, female/male	18/42
BMI, kg/m^2^	27 ± 4
Duration of permanent atrial fibrillation, months	11 (2–121)
CHA_2_DS_2_-VASc score	2.3 ± 1.5
Hypertension	25 (42 %)
Stroke or transient ischemic attack	7 (12 %)
Diabetes Mellitus	3 (5 %)
Chronic obstructive pulmonary disease	3 (5 %)
Current cigarette smoking	3 (5 %)
Alcohol intake, units/week	3.5 (0–35)
Systolic blood pressure, mm Hg	141 ± 18
Diastolic blood pressure, mm Hg	91 ± 10
Heart rate at rest, beats per minute	95 ± 15
Left atrial diameter, long-axis view, mm	50.4 ± 6.6
Left ventricular ejection fraction, %	61.4 ± 7.5
Forced expiratory volume in one second, Liter	2.75 ± 0.83
Forced expiratory volume in one second, % predicted	94.6 ± 16.8
Diffusion capacity of the lung for carbon monoxide, % predicted	87.3 ± 17.3
Hemoglobin, g/dl	14.6 ± 1.2
Estimated glomerular filtration rate, mL/min	77.1 ± 17.6
NT-proBNP, pg/mL	1039 ± 636
*Medication*	
Warfarin	56 (93 %)
Aspirin	4 (7 %)
Angiotensin receptor blocker or angiotensin-converting enzyme inhibitor	22 (37 %)
Diuretics	9 (15 %)
Statins	12 (20 %)
*Rate controlling medication at study entry, before wash-out period*	
Metoprolol	34 (57 %)
Carvedilol	2 (3 %)
Verapamil	11 (18 %)
Diltiazem	1 (2 %)
Digitoxin	8 (13 %)

Values are expressed as mean ± SD, median (range) or frequencies (%). Glomerular filtration rate is estimated from creatinine level, age and gender. Abbreviations: CHA_2_DS_2_-VASc score is a measure of stroke risk in patients with atrial fibrillation, with scores ranging from 0 to 9 and higher scores indicating greater risk; NT-proBNP, N-terminal pro-B-type natriuretic peptide

*SD* standard deviation

**Table 2:** Resting ventricular rate and hs-TnI levels at rest and peak exercise

Treatment	Resting ventricular rate, bpm	Resting systolic blood pressure, mm Hg	Resting diastolic blood pressure, mm Hg	Hs-TnI at rest, ng/L	Hs-TnI at peak exercise, ng/L
Baseline	95 ± 15	141 ± 18	91 ± 10	5.2 (3.8, 8.5)	6.8 (4.5, 9.7)
Diltiazem	77 ± 13^*^	135 ± 13^**^	83 ± 9^*^	4.5 (3.3, 5.9)^*^	5.4 (3.9, 7.4)^*^
Verapamil	82 ± 16^*^	133 ± 15^*^	83 ± 9^*^	4.1 (2.8, 5.6)^*^	5.3 (3.7, 6.8)^*^
Metoprolol	81 ± 15^*^	135 ± 17^**^	86 ± 10^**^	4.1 (2.7, 6.2)^*^	5.2 (3.4, 6.9)^*^
Carvedilol	78 ± 11^*^	132 ± 19^*^	85 ± 10^*^	4.5 (3.0, 6.0)^*^	5.1 (3.8, 6.7)^*^

Values are expressed as mean ± SD or median (25th percentile, 75th percentile) depending on distribution

^*^ *p <* 0.001 compared with baseline

^**^p ≤ 0.01 compared with baseline

bpm, beats per minute

hs-TnI, high-sensitivity troponin I

*SD* standard deviation

**Fig. 3:**
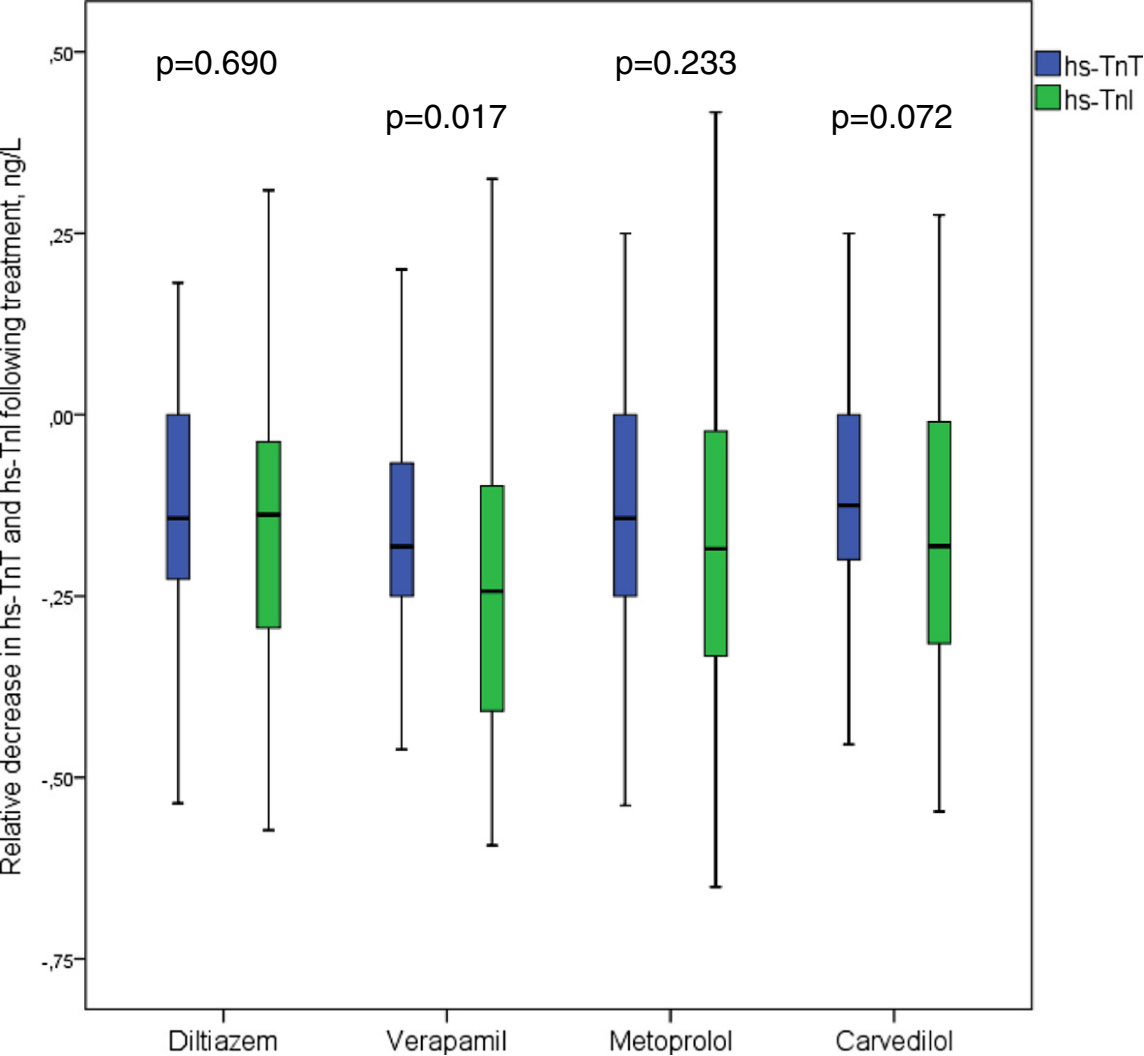
Relative decrease in hs-TnI and hs-TnT levels following treatment. The decline in hs-TnI and hs-TnT levels associated with beta-blocker and calcium channel blocker treatment was similar, except for a larger relative decrease in hs-TnI levels following verapamil treatment (*p =* 0.017) (p-values derived from the Mann–Whitney *U*-test). Center lines show the medians; box limits indicate the 25th and 75th percentiles; whiskers extend 1.5 times the interquartile range from the 25th and 75th percentiles. Abbreviations: hs-TnI, high-sensitivity troponin I; hs-TnT, high-sensitivity troponin T

All drug regimens reduced the resting and the peak exercise levels of hs-TnI compared to baseline (*p <* 0.001 for all; values are given in Table 2 and Fig. 2), with no significant differences between the treatments. Resting hs-TnI and hs-TnT values decreased equally relative to baseline levels, except for verapamil, which reduced hs-TnI more than hs-TnT (*p =* 0.017) (Table 3, Fig. 3). The relative reduction in resting hs-TnI levels did neither correlate to the relative change in resting heart rate nor the relative change in systolic blood pressure. There were no significant correlations between hs-TnI values and symptom severity or frequency, assessed by the SCL questionnaire.

**Table 3:** Hs-TnI and hs-TnT levels at rest

Treatment	Hs-TnI at rest, ng/L	Hs-TnT at rest, ng/L	Decrease in hs-TnI associated with treatment relative to baseline level, ng/L	Decrease in hs-TnT associated with treatment relative to baseline level, ng/L	Decrease in hs-TnI associated with treatment relative to baseline level, %	Decrease in hs-TnT associated with treatment relative to baseline level, %
Baseline	5.2 (3.8, 8.5)	10.0 (7.0, 13.0)				
Diltiazem	4.5 (3.3, 5.9)	9.0 (7.0, 12.0)	0.14 (0.04, 0.30)	0.14 (0.0, 0.23)	14 %	14 %
Verapamil	4.1 (2.8, 5.6)	8.0 (6.0, 11.0)	0.24 (0.09, 0.41)^*^	0.18 (0.05, 0.25)	24 %	18 %
Metoprolol	4.1 (2.7, 6.2)	8.0 (6.0, 10.5)	0.18 (0.02, 0.33)	0.14 (0.0, 0.25)	18 %	14 %
Carvedilol	4.5 (3.0, 6.0)	8.0 (6.0, 12.0)	0.18 (0.01, 0.32)	0.13 (0.0, 0.20)	18 %	13 %

Values are expressed as median (25th percentile, 75th percentile). ^*^ *p <* 0.05 compared with hs-TnT

hs-TnI, high-sensitivity troponin I

hs-TnT, high-sensitivity troponin T

**Fig. 4:**
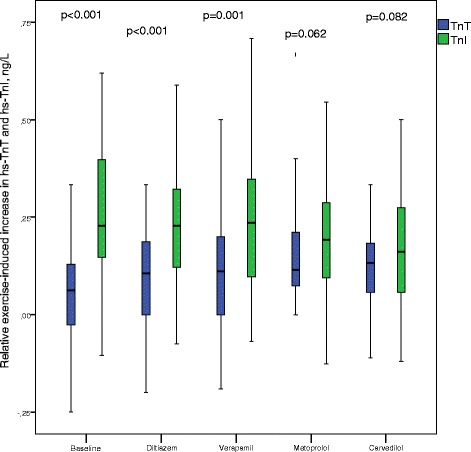
Relative exercise-induced increase in hs-TnI and hs-TnT levels. The relative exercise-induced increase in hs-TnI was significantly larger compared to hs-TnT at baseline (*p <* 0.001) and at treatment with diltiazem (*p <* 0.001) and verapamil (*p =* 0.001) (p-values derived from the Mann–Whitney *U*-test). Center lines show the medians; box limits indicate the 25th and 75th percentiles; whiskers extend 1.5 times the interquartile range from the 25th and 75th percentiles. Abbreviations: hs-TnI, high-sensitivity troponin I; hs-TnT, high-sensitivity troponin T

Levels of hs-TnI increased significantly by exercise testing, both at baseline and with all treatments (*p <* 0.001 for all; values are given in Table 2). The relative exercise-induced increase in hs-TnI was significantly larger compared to hs-TnT at baseline (*p <* 0.001) and at treatment with diltiazem (*p <* 0.001) and verapamil (*p =* 0.001) (Table 4, Fig. 4). There were no significant differences in exercise-induced increase between the two troponin subunits during treatment with beta-blockers. The relative exercise-induced increase in hs-TnI levels was similar in men and in women.

**Table 4:** Hs-TnI and hs-TnT levels at peak exercise

Treatment	Hs-TnI at peak exercise, ng/L	Hs-TnT at peak exercise, ng/L	Increase in hs-TnI in response to exercise relative to resting level, ng/L	Increase in hs-TnT in response to exercise relative to resting level, ng/L	Increase in hs-TnI in response to exercise relative to resting level, %	Increase in hs-TnT in response to exercise relative to resting level, %
Baseline	6.8 (4.5, 9.7)	11.0 (7.0, 14.0)	0.23 (0.14, 0.40)^*^	0.06 (−0.05, 0.13)	23 %	6 %
Diltiazem	5.4 (3.9, 7.4)	9.0 (7.0, 12.0)	0.23 (0.12, 0.32)^*^	0.11 (0.0, 0.19)	23 %	11 %
Verapamil	5.3 (3.7, 6.8)	9.0 (6.0, 12.0)	0.24 (0.10, 0.35)^**^	0.11 (0.0, 0.20)	24 %	11 %
Metoprolol	5.2 (3.4, 6.9)	9.0 (7.0, 12.0)	0.19 (0.09, 0.29)	0.12 (0.07, 0.22)	19 %	12 %
Carvedilol	5.1 (3.8, 6.7)	9.0 (7.0, 13.0)	0.16 (0.06, 0.27)	0.13 (0.04, 0.20)	16 %	13 %

Values are expressed as median (25th percentile, 75th percentile)

^*^ *p <* 0.001 compared with hs-TnT

^**^ *p* ≤ 0.01 compared with hs-TnT

hs-TnI, high-sensitivity troponin I

hs-TnT, high-sensitivity troponin T

**Fig. 5:**
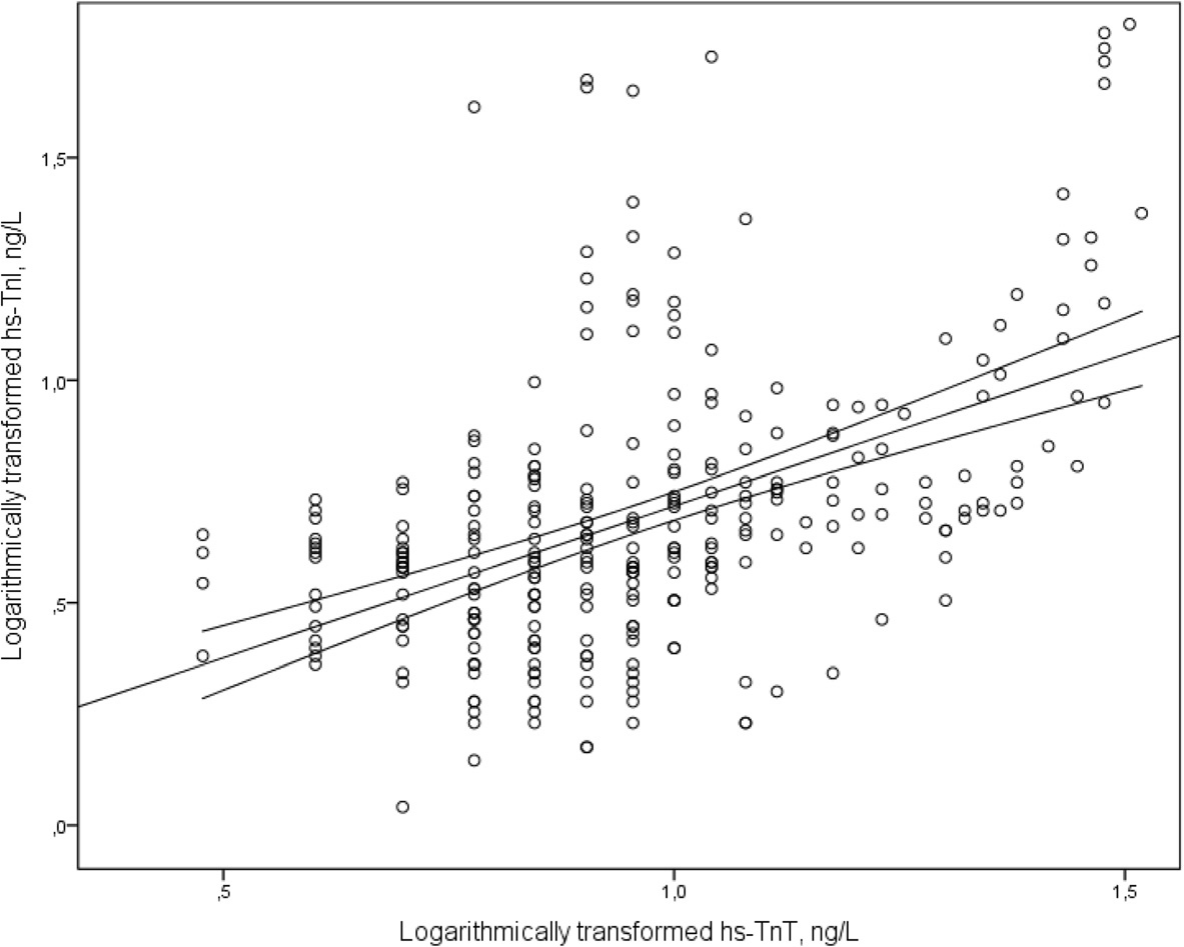
Scatterplot showing the association between baseline levels of logarithmically transformed hs-TnI and hs-TnT. Scatterplot with fitted linear regression line and 95 % confidence interval curves. Regression coefficient was 0.35, 95 % CI (0.28, 0.42), *p <* 0.001, adjusted R^2^ 0.24. Abbreviations: hs-TnI, high-sensitivity troponin I; hs-TnT, high-sensitivity troponin T

The resting levels of hs-TnI and hs-TnT correlated moderately at baseline and at all drug regimens; baseline (r_s_ = 0.582, *p <* 0.001) (Fig. 5), diltiazem (r_s_ = 0.455, *p <* 0.001), verapamil (r_s_ = 0.454, *p <* 0.001), metoprolol (r_s_ = 0.382, *p =* 0.003) and carvedilol ((r_s_ = 0.465, *p <* 0.001).

## Discussion

In the present study of stable patients with permanent AF, normal systolic function and no history of ischemic heart disease, hs-TnI was detectable in all patients. All four rate-controlling drugs reduced hs-TnI levels significantly both at rest and during exercise. The decline in hs-TnI and hs-TnT levels associated with beta-blocker and calcium channel blocker treatment was similar, except for a larger relative decrease in hs-TnI levels following verapamil treatment. The relative exercise-induced increase in hs-TnI was larger than for hs-TnT at baseline and during treatment with calcium channel blockers.

A significant reduction in circulating levels of hs-TnI at rest and at peak exercise was achieved with all four rate-controlling drugs. Although the study drugs have different pharmacodynamic profiles and effects on 24-h heart rate [18], they had similar effects on hs-TnI release. Previous published results from the RATAF study demonstrated similar effects of the four treatments on resting hs-TnT levels, except for a larger relative decrease in hs-TnI levels following verapamil treatment [11]. A causal relationship between heart rate reduction and subsequent lower levels of troponin has been suggested as heart rate may predict troponin I levels in AF patients [12]*.* However, a decreased systolic and diastolic blood pressure has also been associated with lowered levels of cardiac troponins in patients with essential hypertension treated with the calcium channel blocker amlodipine [28]. The changes in hs-TnI levels were not significantly correlated to changes in heart rate in our study, and this may indicate that other mechanisms than heart rate reduction could be of relevance. In addition to the potential effects of blood pressure reduction, patients with subclinical ischemic heart disease may have benefited from the anti-ischemic abilities of the study drugs. Hence, the mechanisms behind the attenuated troponin release induced by rate-controlling drugs in permanent AF need to be investigated further.

The exercise-induced increase in hs-TnI was significant with no differences between the treatments. The relative exercise-induced increase in hs-TnI was significantly larger compared to hs-TnT at baseline and at treatment with calcium channel blockers. The discrepancy could be due to assay properties, as the hs-TnI assay has a lower limit of detection and quantification compared to the hs-TnT assay. Other explanations could be that there are differences in release kinetics between the two molecules, or that the underlying mechanisms responsible for exercise-induced troponin release affect the two subunits differently. While even minor elevations of resting hs-TnT and hs-TnI hold prognostic value in AF, the implications of exercise-induced troponin release in subjects with AF are unknown. A transient, exercise-induced troponin release has been documented in healthy individuals and may represent a physiological response that does not necessarily comprise any deleterious effects to the healthy heart at all [29–31]. Changes in troponin concentrations during exercise stress testing may improve diagnostic accuracy for detection of ischemia in patients with suspected coronary artery disease [32]; however, there are contradictory reports [13, 14].

The correlation between hs-TnI and hs-TnT in this study was relatively moderate, in accordance with observations in an AF cohort [17], and in a population with stable coronary heart disease [15]. The mobilization of troponin T across the cellular membrane of a cardiac myocyte could be attenuated by its larger size and higher molecular weight compared to the smaller troponin I molecule, a proposal which is supported by evidence of troponin I being more sensitive in the setting of acute myocardial infarction [16]. The relatively modest correlation between hs-TnI and hs-TnT may as well be explained by discrepancies in assay properties, permitted by the lack of harmonisation and standardisation between different hs-TnI and hs-TnT platforms [23, 26, 33].

Levels of NT-proBNP correlated with hs-TnT, but not with hs-TnI. All drugs reduced the troponins similarly, whereas beta-blockers increased NT-proBNP levels and calcium channel blockers decreased NT-proBNP levels [20]. The interpretation of elevated NT-proBNP levels in AF is challenging as the arrhythmia itself is associated with an increase in NT-proBNP, and significantly higher cut-off levels are required to diagnose heart failure in AF patients [34]. Heart failure with preserved ejection fraction is prevalent in AF populations [35, 36], and natriuretic peptides may be useful in this setting [37]. However, a diagnosis of diastolic dysfunction by echocardiography is more difficult to make in AF due to the irregular heart rhythm and inherent changes in mitral flow. Tissue Doppler measures could have shed more light on the possible role of diastolic dysfunction in our population; however, such data were not available.

The value of high-sensitivity troponin assays in the clinic is dependent on knowledge of the impact of confounding factors like medications and exercise testing on troponin levels. Interpretation of low-level chronic troponin elevations is a challenge for clinicians, and can only be relieved by better understanding of the mechanisms and factors influencing troponin release. In this perspective, the present study extends the information provided by Ulimoen et al. [11] by showing that rate-control and exercise testing significantly influence hs-TnI levels in an AF population, and that the effects on hs-TnI and hs-TnT are comparable.

## Study limitations

Twenty patients did not fulfil all drug treatment periods and were therefore not included in this analysis. However, these patients had similar baseline characteristics as those who completed all parts of the study. Patients with systolic heart failure were not included in the study; however we have not conducted echocardiographic assessment of diastolic function and cannot exclude the possibility that some patients had heart failure with preserved ejection fraction. Ischemic heart disease was an exclusion criterion in the present study, and we emphasise that our results may not be valid for such patients. The available formulations of the study drugs differ with regard to pharmacokinetic profile, which may have influenced exercise capacity and cardiac troponin levels.

## Conclusions

In the present study of stable patients with permanent AF, normal systolic function and no history of ischemic heart disease, hs-TnI was detectable in all patients. All four rate-controlling drugs reduced hs-TnI levels significantly both at rest and during exercise. The decline in hs-TnI and hs-TnT levels associated with beta-blocker and calcium channel blocker treatment was similar, except for a larger relative decrease in hs-TnI levels following verapamil treatment. The relative increase in hs-TnI during exercise testing was larger than for hs-TnT at baseline and during treatment with calcium channel blockers.

### Availability of data and materials

The data sets will not be publicly available, as the Data Protection Authority approval and patient consent do not allow for such publication.

## Abbreviations

AF: atrial fibrillation
CHA_2_DS_2_-VASc score: a score which assigns one point each for a history of congestive heart failure, hypertension, diabetes mellitus, vascular disease, age 65–74 years and female sex and two points for age ≥ 75 years and prior stroke/transient ischemic attack
hs-TnI: high-sensitivity troponin I
hs-TnT: high-sensitivity troponin T
RATAF study: rate control in Atrial Fibrillation study
SCL: symptom checklist—frequency and severity
